# Pandemic-related disruption and post-pandemic recovery of dental service production in Brazilian Primary Health Care, 2015-2025

**DOI:** 10.31744/einstein_journal/2026AO2527

**Published:** 2026-09-01

**Authors:** Laura Jackeline García Rincón, Ana Luiza Felizardo David, Raphael Daniel Rodrigues, Giovanna Oliveira Souza, Emanuelle Thomazini Zambom, Caio Possi Yazaki, Gabriela Fulanetto Crestana, Lorrayne Belotti

**Affiliations:** 1 CEPPAR Hospital Israelita Albert Einstein São Paulo SP Brazil Centro de Estudos, Pesquisa e Práticas em Atenção Primária à Saúde e Redes (CEPPAR), Hospital Israelita Albert Einstein, São Paulo, SP, Brazil.; 2 Faculdade Israelita de Ciências da Saúde Albert Einstein Hospital Israelita Albert Einstein São Paulo SP Brazil Faculdade Israelita de Ciências da Saúde Albert Einstein, Hospital Israelita Albert Einstein, São Paulo, SP, Brazil.

**Keywords:** Primary health care, Oral health, COVID-19, Pandemics, Health services accessibility, Health services research

## Abstract

**Objective:**

To analyze changes in dental service production in Brazilian Primary Health Care from 2015 to 2025 and characterize pandemic-associated disruption and post-pandemic recovery by procedure type and Federative Unit.

**Methods:**

This ecological interrupted time-series study used secondary data on primary health care dental procedures recorded in the Brazilian Unified Health System. Six indicators were evaluated: scheduled consultations, health promotion and prevention activities, and surgical, endodontic, periodontal, and restorative procedures. Annual rates per 100,000 inhabitants were calculated. Three periods were defined: pre-pandemic (2015-2019), pandemic (2020-2021), and recovery (2022-2025). National percentage changes were calculated using selected annual rates, whereas the proportion of the pandemic-related decline recovered was calculated using period means. Interrupted time-series analyses were performed using segmented regression with Newey-West standard errors.

**Results:**

Dental service production declined during the pandemic across all indicators, and mean recovery-period levels remained below pre-pandemic means. The largest reductions between 2019 and 2021 occurred in health promotion and prevention activities (-79.6%), periodontal procedures (-62.5%), scheduled consultations (-58.8%), and surgical procedures (-53.8%). Recovery remained incomplete across all indicators, ranging from 7.6% for scheduled consultations to 68.8% for restorative procedures. Total procedure rates showed a significant pre-pandemic annual decline of 21.4% (95%CI=-24.9% to -17.7%), an immediate reduction of 56.1% in 2020 (95%CI=-71.4% to -32.6%), and a positive post-2020 annual change of 14.8% (95%CI=1.2% to 30.2%). Marked territorial heterogeneity was observed in both the magnitude of decline and the pattern of recovery.

**Conclusion:**

Dental service production in Brazilian Primary Health Care declined substantially during the COVID-19 pandemic and recovered only partially during 2022-2025, with persistent territorial disparities in recorded service production.

## HIGHLIGHTS

■ Dental service production declined sharply during the COVID-19 pandemic.

■ Recovery remained incomplete across all dental service indicators during 2022–2025.

■ Oral health promotion and prevention activities showed the largest reductions.

■ Recovery patterns varied markedly across Brazilian Federative Units.

## In Brief

The COVID-19 pandemic was associated with a substantial decline in dental service production in Brazilian Primary Health Care. Although production increased after 2020, recovery remained incomplete through 2025 and varied considerably among Federative Units, indicating persistent regional disparities in oral health service delivery.

## Graphical abstract

**Figure:**
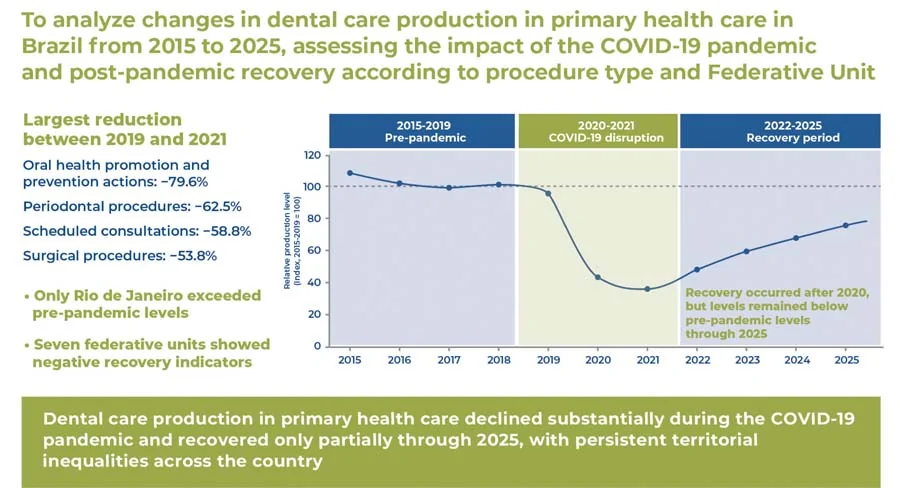

## INTRODUCTION

Primary Health Care (PHC) forms the foundation of Brazil’s Public Health System (SUS - *Sistema Único de Saúde*), one of the world’s largest universal health systems, supported by an extensive service network. Guided by the principles of equity, universality, and comprehensive care, PHC serves as the main entry point to the health system and coordinates care across services, thereby supporting efforts to reduce health inequalities. Its nationwide reach supports both routine care and coordinated responses during public health emergencies, as demonstrated during the COVID-19 pandemic.^(1-3)^

Within this framework, oral health is an integral component of comprehensive and person-centered care because of its close relationship with systemic health. Its integration into PHC may influence quality of life through effects on nutrition, communication, self-esteem, and social well-being. Historically, the expansion of Oral Health Teams (OHTs) in Brazil marked an important step toward broadening access to dental care and shifting the traditional treatment-focused model toward health surveillance and promotion.^(4,5)^ Despite the expansion of services through the growth of Oral Health Teams and the establishment of Dental Specialty Centers, improvements in access, service effectiveness, and epidemiological indicators have been uneven and, in some settings, limited. In some settings, service delivery continued to resemble the traditional care model, with only modest improvements in oral health indicators.^(6)^

Monitoring dental service production through information systems such as Outpatient Information System of the Brazilian Public Health System (SIA/SUS - *Sistema de Informações Ambulatoriais do Sistema Único de Saúde*) is important for evaluating service organization and performance. Analyses of service profiles and temporal trends can inform public policy planning and monitoring, particularly when different categories of dental procedures are examined separately.^(7,8)^ Regional differences in production patterns reveal persistent inequalities in access, infrastructure, and management capacity, underscoring challenges to equitable oral health care across Brazil.

However, the COVID-19 pandemic prompted a rapid reorganization of service networks,^(3)^ requiring PHC services to adapt workflows and priorities in response to the crisis.^(9)^ As a containment measure, official guidelines recommended suspending elective appointments and maintaining only urgent care nationwide.^(10)^ In dentistry, service disruption was particularly pronounced because care requires close physical proximity and frequently involves aerosol-generating procedures, increasing the risk of viral transmission.^(11,12)^ This scenario substantially reduced procedure volumes, while the redeployment of dentists to frontline roles may have further constrained service delivery and contributed to deferred demand.^(11,13,14)^

Although previous studies have documented the immediate effects of the crisis during 2020-2021,^(11,13,15)^ longer-term recovery patterns remain insufficiently characterized. Few studies have examined dental service production over an extended period to characterize healthcare recovery after pandemic-related disruption and deferred care. Evidence is also limited regarding differences among procedure categories and the heterogeneity of recovery across Federative Units, which may reflect local management capacity and longstanding territorial inequalities.

## OBJECTIVE

This study aimed to analyze changes in dental service production in Brazilian Primary Health Care from 2015 to 2025. Specifically, it evaluated pandemic-associated changes and post-pandemic recovery patterns across dental procedure indicators and Federative Units.

## METHODS

### Type of study

This ecological study used aggregated secondary data and combined a national interrupted time-series analysis with descriptive analyses at the Federative Unit level. At the national level, annual dental service production rates were analyzed from 2015 through 2025. For the descriptive analyses, each Federative Unit-year combination constituted the unit of analysis, allowing characterization of temporal variation and territorial heterogeneity in PHC dental service production.

Ethics approval was not required because the study used only publicly available, aggregated secondary data.

### Study period

Data from 2015 through 2025 were analyzed, providing 11 annual observations. For descriptive purposes, the study period was divided into three epidemiological phases: pre-pandemic, pandemic, and recovery. Annual rates were used to reduce short-term reporting fluctuations, improve comparability across years and Federative Units, and characterize long-term trends.

The pre-pandemic period was defined as 2015-2019, representing the baseline phase before the onset of the COVID-19 pandemic and the associated disruption of routine PHC services.

The pandemic period was defined as 2020-2021, when health services in Brazil experienced the greatest disruption, including the suspension or restriction of elective dental procedures, reorganization of care pathways, and prioritization of urgent and emergency care.

The recovery period was defined operationally as 2022-2025, corresponding to the resumption and reorganization of PHC dental service delivery after the most restrictive phase of the pandemic. Although the international public health emergency ended later, 2022 was selected as the beginning of the recovery period because routine and elective dental services had already begun to resume after the acute phase of disruption.

For the interrupted time-series analysis, 2020 was defined as the interruption point, corresponding to the onset of the COVID-19 pandemic and the introduction of major restrictions on dental service provision.

### Data source

Data on dental service production were extracted from the SIA/SUS. Annual population estimates for each Federative Unit were obtained from the Brazilian Institute of Geography and Statistics (IBGE - *Instituto Brasileiro de Geografia e Estatistica*).

Data were extracted from SIA/SUS in March 2026. The 2025 dataset included procedures recorded from January through December 2025. Because SIA/SUS outpatient production records may be submitted or revised for up to three months after a procedure is performed, the 2025 data were considered consolidated for routine processing purposes at the time of extraction. Nevertheless, because administrative databases may undergo subsequent correction or exceptional reprocessing, analyses involving 2025 were interpreted cautiously.

Dental procedures were grouped according to codes from the Management System for the Table of Procedures, Medications, Orthotics, Prosthetics, and Special Materials (SIGTAP - *Sistema de Gerenciamento da Tabela de Procedimentos, Medicamentos e OPM do SUS*) into six analytical indicators: scheduled consultations, oral health promotion and prevention activities, and surgical, endodontic, periodontal, and restorative procedures (Table 1). The grouping was defined based on the official SIGTAP procedure-table structure and the conceptual relevance of each category to PHC dental service production.

**Table 1:** SIGTAP procedures included in each analytical group

Procedures	Specifications	SIGTAP codes
Scheduled consultation	03.01.01 Consultations	03.01.01.015-3 - Scheduled initial dental consultation
Oral health promotion and prevention activities	01. Health promotion and prevention activities 01. Collective and individual activities 02. Oral health	01.01.02.001-5 - Collective topical fluoride-gel application
		01.01.02.002-3 - Collective fluoride mouth-rinse program
		01.01.02.003-1 - Supervised toothbrushing program (group activity)
		01.01.02.004-0 - Collective oral examination for epidemiological purposes
		01.01.02.005-8 - Application of a cariostatic agent (per tooth)
		01.01.02.006-6 - Pit-and-fissure sealant application (per tooth)
		01.01.02.007-4 - Topical fluoride application (individual session)
		01.01.02.008-2 - Dental plaque-disclosure procedure
		01.01.02.009-0 - Temporary sealing of a dental cavity
		01.01.02.010-4 - Oral hygiene instruction
		01.01.02.011-2 - Collective oral cancer prevention activity
		01.01.02.012-0 - Dental prosthesis hygiene instruction
Restorative procedures	03. Clinical procedures 07. Dental treatments 01. Dentistry	03.07.01.001-5 - Pulp capping
		03.07.01.002-3 - Primary tooth restoration (revoked in 2020)
		03.07.01.003-1 - Anterior permanent tooth restoration with composite resin
		03.07.01.004-0 - Posterior permanent tooth restoration (revoked in 2020)
		03.07.01.006-6 - Initial management of a traumatized tooth
		03.07.01.007-4 - Atraumatic restorative treatment (ART)
		03.07.01.008-2 - Posterior primary tooth restoration with composite resin
		03.07.01.009-0 - Posterior primary tooth restoration with amalgam
		03.07.01.010-4 - Posterior primary tooth restoration with glass-ionomer cement
		03.07.01.011-2 - Anterior primary tooth restoration with composite resin
		03.07.01.012-0 - Posterior permanent tooth restoration with composite resin
		03.07.01.013-9 - Posterior permanent tooth restoration with amalgam
		03.07.01.014-7 - Behavioral management of persons with disabilities
		03.07.01.015-5 - Behavioral management of children
Endodontic procedures	03. Clinical procedures 07. Dental treatments 02. Endodontics	03.07.02.001-0 - Access to the dental pulp and intracanal medication (per tooth)
		03.07.02.002-9 - Temporary dressing with or without biomechanical preparation
		03.07.02.007-0 - Dental pulpotomy
Periodontal procedures	03. Clinical procedures 07. Dental treatments 03. Clinical periodontics	03.07.03.001-6 - Supragingival scaling, root planing, and polishing (per sextant; revoked in May 2016)
		03.07.03.002-4 - Subgingival scaling and root planing (per sextant)
		03.07.03.004-0 - Prophylaxis/dental plaque removal
		03.07.03.005-9 - Supragingival scaling, root planing, and polishing (per sextant)
		03.07.03.006-7 - Treatment of acute necrotizing ulcerative gingivitis (ANUG)
		03.07.03.007-5 - Treatment of oral mucosal lesions
		03.07.03.008-3 - Treatment of pericoronitis
Surgical procedures	04. Surgical procedures 14. Oral and maxillofacial surgery 02. Oral surgery	04.14.02.012-0 - Extraction of a primary tooth
		04.14.02.013-8 - Extraction of a permanent tooth
		04.14.02.017-0 - Glossorrhaphy
		04.14.02.035-9 - Surgical management of oral hemorrhage
		04.14.02.038-3 - Treatment of alveolitis (dry socket)
		04.14.02.040-5 - Ulotomy/ulectomy
		04.14.02.043-0 - Extraction of a supernumerary tooth

Source: http://sigtap.datasus.gov.br/tabela-unificada/app/sec/inicio.jsp

The oral health promotion and prevention indicator included both collective and individual preventive activities. Accordingly, this indicator was interpreted separately from the clinical procedure indicators because collective activities, such as supervised toothbrushing, fluoride mouth-rinse programs, and group-based oral health initiatives, may have been disproportionately affected by school closures and restrictions on group activities during the pandemic.

SIGTAP codes were reviewed across the study period to identify procedures that were revoked, replaced, or introduced during the series. When procedure codes were revoked but continued to represent the same analytical group, they were retained for the years in which they remained valid and harmonized within the corresponding category. This approach was intended to preserve historical comparability across procedure groups and reduce the likelihood that observed temporal changes reflected coding revisions rather than actual changes in service production.

### Construction of variables

The analytical variables were derived from annual totals of PHC dental service production and the corresponding population estimates, as described in table 2. The table presents the operational definition, calculation method, and interpretation of each variable, including absolute measures, rates per 100,000 inhabitants, measures of central tendency and dispersion, percentage changes, and the proportion of decline recovered across periods. This structure was intended to improve methodological transparency and analytical reproducibility.

**Table 2:** Operational definitions and calculation methods for study variables, Brazil, 2015-2025

Indicator	Description	Calculation method	Interpretation
Total annual procedures	Annual number of dental procedures performed in PHC in each Federative Unit or nationally	Sum of the six indicators – scheduled consultations, health promotion and prevention activities, and surgical, endodontic, periodontal, and restorative procedures – for each Federative Unit and year	Represents the absolute volume of dental service production in PHC
Rate per 100,000 inhabitants	Dental service production standardized to population size	(Total annual procedures ÷ resident population in the same year) × 100,000	Allows comparison across Federative Units and over time after accounting for population size
Median Federative Unit rate by period	Measure of the central tendency of rates per 100,000 inhabitants across Federative Units within each epidemiological period	Median of all Federative Unit-level rates within each epidemiological period (2015-2019, 2020-2021, and 2022-2025), with each Federative Unit-year combination treated as a separate observation	Represents the typical value of service production across Federative Units in each phase of the series
Q25-Q75 interval	Measure of dispersion in Federative Unit rates within each period	Range between the 25th and 75th percentiles (Q25-Q75) of Federative Unit rates within each period	Indicates heterogeneity among Federative Units and summarizes the dispersion of rates within each period
Mean rate by epidemiological period	Average level of dental service production within each epidemiological period	Mean of the annual national rates within the pre-pandemic (2015-2019), pandemic (2020-2021), and recovery (2022-2025) periods	Reduces the influence of individual annual fluctuations and summarizes the average production level during each phase of the series
Percentage changes	Relative change between two selected years	[(Final-year rate - initial-year rate) ÷ initial-year rate] × 100, applied to 2019-2021, 2021-2023, and 2023-2025	Negative values indicate a decline, positive values indicate an increase, and the magnitude reflects the extent of relative change
Percentage of decline recovered	Proportion of the pandemic-period decline regained during the recovery period	Decline=pre-pandemic mean - pandemic mean; Gain=recovery mean - pandemic mean; Percentage recovered=(gain ÷ decline) × 100, when the decline was greater than 0	A value of 100% indicates a return to the pre-pandemic mean; values above 100% indicate that the recovery-period mean exceeded the pre-pandemic mean; values between 0% and 100% indicate partial recovery; and negative values indicate that the recovery-period mean remained below the pandemic-period mean

### Data analyses

As described above, annual rates per 100,000 inhabitants were calculated for each dental service indicator at the national and Federative Unit levels to enable comparisons over time and across populations of different sizes. For descriptive analyses, the study period was divided into the same three predefined epidemiological phases: pre-pandemic (2015-2019), pandemic (2020-2021), and recovery (2022-2025). Federative Unit-level rates were summarized for each period using medians and Q25-Q75 intervals because of distributional asymmetry and territorial heterogeneity.

National percentage changes were calculated for three selected intervals: 2019-2021, representing the change from the final pre-pandemic year to the second pandemic year; 2021-2023, representing the early post-pandemic change; and 2023-2025, representing the most recent change. The percentage of decline recovered was calculated using period means as follows: decline=pre-pandemic mean - pandemic mean; gain=recovery mean - pandemic mean; and percentage recovered=(gain ÷ decline) × 100, when the decline was greater than 0.

A value of 100% indicated a return to the pre-pandemic mean; values above 100% indicated that the recovery-period mean exceeded the pre-pandemic mean; values between 0% and 100% indicated partial recovery; and negative values indicated that the recovery-period mean remained below the pandemic-period mean.

A descriptive spatial analysis was performed using thematic maps for each Federative Unit. The maps displayed the percentage change between the mean pre-pandemic and pandemic rates and the percentage of decline recovered during the recovery period. These analyses characterized territorial heterogeneity in pandemic-associated declines and post-pandemic recovery.

To assess temporal changes in national dental service production, an interrupted time-series analysis was performed using segmented regression models. The year 2020 was defined as the interruption point, corresponding to the onset of the COVID-19 pandemic and the introduction of major restrictions on dental service provision in Brazil. For each dental service indicator, the annual national rate per 100,000 inhabitants was modeled as the outcome.

The segmented regression model included terms for time, the post-interruption period, and time after the interruption, allowing estimation of the pre-pandemic annual trend, the immediate level change in 2020, and the post-2020 change in slope. The model was specified as:


$$\log\left(Y_{t}\right)=\beta_{0}+\beta_{1}T_{t}+\beta_{2}X_{t}+\beta_{3}Z_{t}+\varepsilon_{t}$$

where Y_t_ represents the national annual rate per 100,000 inhabitants in year t; T_t_ represents the time elapsed since the beginning of the series; X_t_ is an indicator variable for the post-interruption period, coded 0 before 2020 and 1 from 2020 onward; and Z_t_ represents the time elapsed after the interruption, coded 0 through 2020 and sequentially thereafter. In this model, β_1_ estimated the pre-pandemic annual trend, β_2_ estimated the immediate level change in 2020, and β_3_ estimated the change in slope after the interruption. The post-2020 annual trend was estimated from the combined β_1_ and β_3_ coefficients. For interpretation, the combined coefficient (β_1_ + β_3_) was exponentiated and expressed as a percentage annual change, with confidence intervals derived from the corresponding variance-covariance matrix.Models were estimated using ordinary least squares segmented regression with heteroskedasticity- and autocorrelation-consistent Newey-West standard errors to account for potential serial correlation between adjacent annual observations and nonconstant residual variance. The natural logarithm of the annual rates was used as the dependent variable, allowing the coefficients to be interpreted as relative changes. Regression coefficients were exponentiated and expressed as percentage changes with 95% confidence intervals. Residual diagnostics were examined to assess model fit, autocorrelation, heteroskedasticity, and influential observations, with no major concerns identified.

All analyses were performed in R version 4.5.3 using packages for data management, statistical modeling, and visualization.

## ❚RESULTS

From 2015 through 2025, dental service production rates in PHC varied across indicators, epidemiological periods, and Federative Units. Federative Unit-level rates declined during the pandemic period across all indicators, and recovery-period medians remained below their corresponding pre-pandemic medians (Figure 1).

**Figure 1:**
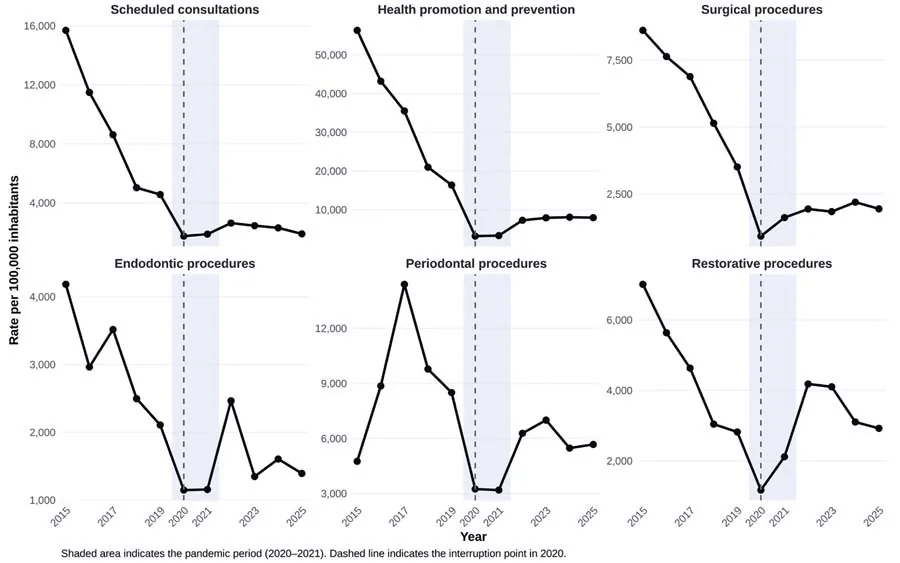
Dental service production in Primary Health Care, Brazil, 2015-2025

The median rate of scheduled consultations decreased from 7,532.6 per 100,000 inhabitants during the pre-pandemic period to 1,002.1 during the pandemic period and 1,008.7 during the recovery period. Health promotion and prevention activities had the highest pre-pandemic median rate, which decreased from 22,761.6 to 1,484.6 and subsequently increased to 2,596.5 per 100,000 inhabitants. The median surgical-procedure rate decreased from 4,005.2 to 657.1 and subsequently increased to 790.9 per 100,000 inhabitants during the recovery period. The median endodontic-procedure rate decreased from 1,553.3 to 626.1 and then increased to 812.5 per 100,000 inhabitants. The median periodontal-procedure rate decreased from 6,802.1 to 1,672.9 and then increased to 2,698.9, whereas the median restorative-procedure rate decreased from 3,936.4 to 655.4 and then increased to 1,744.0 per 100,000 inhabitants (Table 3).

**Table 3:** Median dental service production rates per 100,000 inhabitants across the three study periods

Indicator	Pre-pandemic (2015-2019)	Pandemic (2020-2021)	Recovery (2022-2025)
Scheduled consultations	7532.6 (3794.8-12592.9)	1002.1 (394.1-1958.5)	1008.7 (319.3-2466.2)
Health promotion and prevention activities	22761.6 (9777.1-45213.9)	1484.6 (703.5-3641.8)	2596.5 (1339.5-7152.0)
Surgical procedures	4005.2 (2001.3-8537.1)	657.1 (460.0-1077.2)	790.9 (517.5-1621.5)
Endodontic procedures	1553.3 (1020.7-3041.9)	626.1 (273.1-1098.8)	812.5 (529.7-1289.4)
Periodontal procedures	6802.1 (2846.1-11676.7)	1672.9 (676.3-3179.7)	2698.9 (1225.8-4702.9)
Restorative procedures	3936.4 (1658.8-5445.6)	655.4 (350.5-1635.5)	1744.0 (800.8-3428.1)

Values are medians across Federative Units, with Q25-Q75 intervals shown in parentheses.

At the national level, all indicators declined between 2019 and 2021 (Table 4). The largest reductions occurred in health promotion and prevention activities (-79.6%), periodontal procedures (-62.5%), scheduled consultations (-58.8%), and surgical procedures (-53.8%). Endodontic and restorative procedures also declined, although the relative reductions were smaller (-45.1% and -24.9%, respectively).

**Table 4:** National rates, percentage changes, and recovery in dental service production in Primary Health Care, Brazil, 2019-2025.

Indicator	2019 rate*	2021 rate*	2023 rate*	2025 rate*	Change, 2019-2021^**†**^ %	Change, 2021-2023^**‡**^ %	Change, 2023-2025^**§**^ %	Decline recovered, %^‖^ %
Scheduled consultations	4565.3	1882.7	2455.0	1902.9	-58.8	+30.4	-22.5	7.6
Health promotion and prevention	16379.2	3343.0	7923.3	7962.3	-79.6	+137.0	+0.5	15.3
Surgical procedures	3511.3	1621.5	1845.1	1948.2	-53.8	+13.8	+5.6	14.8
Endodontic procedures	2108.5	1156.9	1348.3	1394.0	-45.1	+16.5	+3.4	31.1
Periodontal procedures	8504.2	3190.3	7001.6	5677.2	-62.5	+119.5	-18.9	50.6
Restorative procedures	2823.0	2120.2	4104.5	2926.3	-24.9	+93.6	-28.7	68.8

*Rate per 100,000 inhabitants, calculated by dividing the national annual total by the corresponding national population; ^†^ percentage change between 2019 and 2021, calculated using annual national rates. This measure represents the change from the final pre-pandemic year to the second pandemic year; ^‡^ percentage change between 2021 and 2023, calculated using annual national rates. This measure represents the early post-pandemic change; ^§^ percentage change between 2023 and 2025, calculated using annual national rates. This measure represents the most recent observed change; *‖*percentage of decline recovered, calculated using period means as follows: decline=pre-pandemic mean rate (2015-2019) - pandemic mean rate (2020-2021); gain=recovery mean rate (2022-2025) - pandemic mean rate (2020-2021); and percentage recovered=(gain ÷ decline) × 100 when the decline was greater than 0. A value of 100% indicates a return to the pre-pandemic mean; values above 100% indicate that the recovery-period mean exceeded the pre-pandemic mean; values between 0% and 100% indicate partial recovery; and negative values indicate that the recovery-period mean remained below the pandemic-period mean.

Between 2021 and 2023, all indicators increased, with the largest relative increases observed in health promotion and prevention activities (+137.0%), periodontal procedures (+119.5%), and restorative procedures (+93.6%). However, these increases were not sustained across all indicators. Between 2023 and 2025, scheduled consultations (-22.5%), periodontal procedures (-18.9%), and restorative procedures (-28.7%) declined again, whereas health promotion and prevention activities (+0.5%), surgical procedures (+5.6%), and endodontic procedures (+3.4%) showed modest increases.

Based on period means, the percentage of decline recovered remained below 100% across all indicators, indicating incomplete recovery relative to the pre-pandemic mean. Restorative procedures showed the highest percentage of decline recovered (68.8%), followed by periodontal procedures (50.6%) and endodontic procedures (31.1%). Lower recovery percentages were observed for health promotion and prevention activities (15.3%), surgical procedures (14.8%), and scheduled consultations (7.6%).

The maps showed marked territorial heterogeneity in both the pandemic-period decline and recovery through 2025 (Figures 2 and 3).

Figure 2 shows substantial heterogeneity in the decline in dental service production across Federative Units between the pre-pandemic and pandemic periods. The smallest declines occurred in Amapá, Rio de Janeiro, Pará, and Roraima, whereas the largest occurred in Piauí, Alagoas, the Federal District, Amazonas, Rio Grande do Norte, Acre, and Rondônia, all of which recorded declines exceeding 90%.

**Figure 2:**
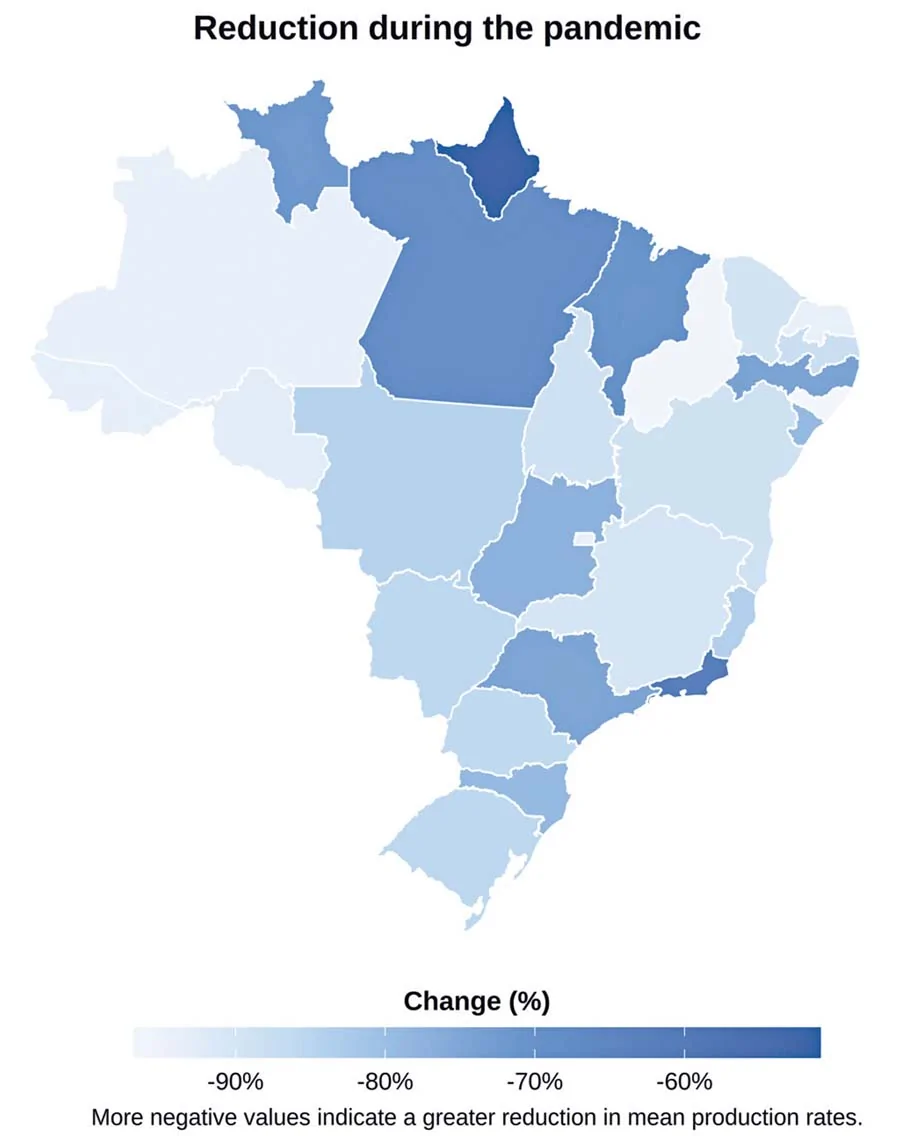
Decline in the dental service production rate by Federative Unit: pre-pandemic period (2015-2019) versus pandemic period (2020-2021)

Figure 3 shows that recovery through 2025 also varied markedly across Federative Units. Rio de Janeiro was the only Federative Unit in which the recovery-period mean exceeded the pre-pandemic mean, whereas Santa Catarina, Rondônia, Acre, Paraná, Bahia, São Paulo, and Paraíba showed comparatively greater partial recovery. In contrast, several Federative Units showed little or no recovery, and seven – Ceará, Roraima, Espírito Santo, Tocantins, Pará, Sergipe, and Maranhão – had negative recovery percentages, indicating that mean production during 2022-2025 remained below the pandemic-period mean.

**Figure 3:**
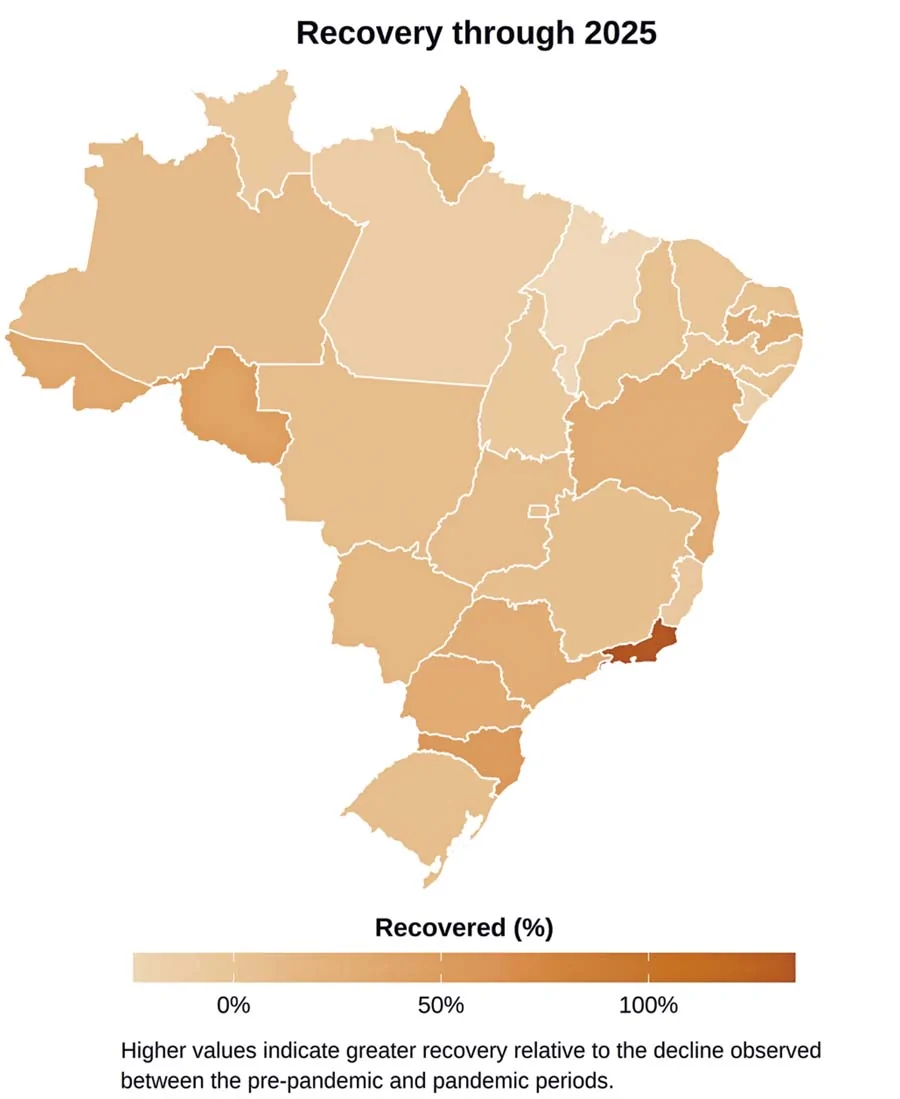
Percentage of decline recovered in the mean total dental service production rate by Federative Unit during the recovery period (2022-2025)

The interrupted time-series analysis showed a significant pre-pandemic decline in national dental service production rates for most indicators. Periodontal procedures were an exception, showing a positive but non-significant pre-pandemic trend (+13.4%; 95%CI=-12.0% to 46.3%; p=0.280) (Table 5). Total procedure rates decreased by 21.4% annually before the pandemic (95%CI=-24.9% to -17.7%; p<0.001). In 2020, there was an immediate level reduction of 56.1% (95%CI=-71.4% to -32.6%; p=0.003), followed by a positive change in slope after the interruption of 46.1% (95%CI=27.2% to 67.8%; p<0.001). The estimated post-2020 annual change was 14.8% (95%CI=1.2% to 30.2%; p=0.036).

**Table 5:** Interrupted time-series analysis of national dental service production rates per 100,000 inhabitants, Brazil, 2015-2025

Indicator	Pre-pandemic annual change, % (95% CI); p value	Immediate level change in 2020, % (95% CI); p value	Change in slope after 2020, % (95% CI); p value	Post-2020 annual change, % (95% CI); p value
Scheduled consultations	-28.1 (-31.4; -24.6); <0.001	-34.3 (-56.5; -0.7); 0.047	42.9 (29.7; 57.4); <0.001	2.8 (-6.9; 13.5); 0.531
Health promotion and prevention	-27.3 (-30.0; -24.5); <0.001	-71.1 (-80.6; -56.8); <0.001	69.5 (49.7; 91.9); <0.001	23.2 (9.5; 38.6); 0.004
Surgical procedures	-19.7 (-25.5; -13.3); <0.001	-61.2 (-73.9; -42.4); <0.001	41.8 (19.5; 68.2); 0.002	13.9 (0.3; 29.3); 0.046
Endodontic procedures	-14.3 (-18.1; -10.3); <0.001	-28.8 (-54.9; 12.4); 0.122	21.3 (6.9; 37.5); 0.008	3.9 (-7.1; 16.2); 0.441
Periodontal procedures	13.4 (-12.0; 46.3); 0.280	-72.0 (-88.2; -33.9); 0.010	0.3 (-25.1; 34.3); 0.979	13.8 (1.1; 28.1); 0.037
Restorative procedures	-21.6 (-24.2; -19.0); <0.001	-14.3 (-64.1; 104.4); 0.687	50.1 (19.4; 88.7); 0.004	17.7 (-7.3; 49.3); 0.151
Total procedures	-21.4 (-24.9; -17.7); <0.001	-56.1 (-71.4; -32.6); 0.003	46.1 (27.2; 67.8); <0.001	14.8 (1.2; 30.2); 0.036

Models were estimated using segmented ordinary least squares regression with heteroskedasticity- and autocorrelation-consistent Newey-West standard errors. The dependent variable was the natural logarithm of annual national rates per 100,000 inhabitants. The interruption point was 2020. Percentage changes and 95% confidence intervals were obtained by exponentiating the model coefficients. The post-2020 annual change corresponds to the combined coefficients for the pre-pandemic annual trend and the post-2020 slope change on the log scale and subsequently transformed to percentage values. Given the limited number of annual observations, these estimates should be interpreted cautiously as temporal associations rather than causal effects.

The largest immediate reductions in 2020 occurred in periodontal procedures (-72.0%; 95%CI=-88.2% to -33.9%; p=0.010), health promotion and prevention activities (-71.1%; 95%CI=-80.6% to -56.8%; p<0.001), and surgical procedures (-61.2%; 95%CI=-73.9% to -42.4%; p<0.001). Scheduled consultations also showed an immediate reduction in 2020 (-34.3%; 95%CI= -56.5% to -0.7%; p=0.047). Immediate level changes were not statistically significant for endodontic and restorative procedures.

After 2020, positive slope changes were observed for most indicators, particularly health promotion and prevention activities, restorative procedures, surgical procedures, scheduled consultations, and total procedures. The estimated post-2020 annual change was significant for health promotion and prevention activities (+23.2%; 95%CI=9.5% to 38.6%; p=0.004), surgical procedures (+13.9%; 95%CI=0.3% to 29.3%; p=0.046), periodontal procedures (+13.8%; 95%CI=1.1% to 28.1%; p=0.037), and total procedures (+14.8%; 95%CI=1.2% to 30.2%; p=0.036). For scheduled consultations, endodontic procedures, and restorative procedures, the post-2020 annual changes were not statistically significant.

## DISCUSSION

This study analyzed dental service production in PHC within the SUS before, during, and after the COVID-19 pandemic in Brazil. The time-series analysis identified three interconnected patterns: a pre-pandemic downward trend from 2015 to 2019, an immediate and widespread decline in 2020, and a subsequent but incomplete and heterogeneous recovery after the most disruptive phase of the pandemic. Numerical reductions were observed across all indicators in 2020, with the largest statistically significant immediate declines occurring in health promotion and prevention activities, periodontal procedures, and surgical procedures. Although production increased in subsequent years, mean levels during 2022-2025 remained below the corresponding pre-pandemic means across all indicators. Restorative procedures showed the greatest proportion of decline recovered, although recovery remained incomplete when assessed using period means.

The pre-pandemic downward trend suggests that the vulnerability observed during the health crisis preceded 2020. Periodontal procedures constituted an exception, showing a positive but non-significant pre-pandemic trend, indicating a distinct pre-pandemic pattern relative to the other indicators. This pattern is consistent with reports of progressive reductions in federal funding for the National Oral Health Policy (PNSB) from 2017 onward, in the context of fiscal austerity and Constitutional Amendment No. 95/2016, which limited federal public spending.^(16-18)^ Previous time-series studies likewise reported declining oral health service-production indicators during this period despite the relative maintenance of service implementation, suggesting that the 2020 disruption may have intensified pre-existing structural vulnerabilities in Brazilian dental PHC.^(19)^

The decline observed between 2019 and 2021 was consistent with national and international evidence documenting pandemic-related disruptions in health service delivery. A systematic review by Moynihan et al.^(15)^ reported a substantial global reduction in health service use during the first year of the COVID-19 pandemic. In dentistry, Meng et al.^(14)^ highlighted the occupational risks associated with aerosol generation, which contributed to the suspension of elective care in several countries. Similarly, Guo et al.^(20)^ and Cunha et al^.(21)^ reported declines in dental service use during the initial phase of the pandemic.

The resumption of dental service production from 2022 onward varied across procedure categories. Restorative procedures showed the strongest recovery, with the 2023 rate exceeding the 2019 level, although recovery remained incomplete when assessed using period means. One possible explanation is the accumulation of unmet need during the pandemic, when substantial volumes of elective care were postponed and may have added to incident demand after services reopened. A similar pattern was reported in US health centers, where restorative and surgical dental care recovered more rapidly than preventive care and exceeded pre-pandemic levels during the first months after services fully resumed.^(22)^

The declines in scheduled consultations, periodontal procedures, and restorative procedures between 2023 and 2025 indicate that recovery was not sustained uniformly across indicators. This pattern may reflect changes in demand after some deferred care was addressed, together with persistent structural barriers to service provision, such as limited OHTs capacity, professional turnover, and funding constraints. World Health Organization reports on the continuity of essential health services indicate that many health systems experienced unstable recovery after the acute pandemic phase, with initial gains followed by further operational challenges.^(23)^ These findings support continued monitoring and structural measures to promote sustainable dental service delivery.

The observed territorial heterogeneity was a central finding and should be interpreted in the context of longstanding inequalities in the organization and capacity of oral health services in Brazil.^(24)^ Although all Federative Units recorded declines in dental service production during the pandemic, the magnitude of decline and, particularly, the extent of recovery varied considerably across the country. This pattern is consistent with evidence that implementation and consolidation of the National Oral Health Policy have occurred unevenly across Brazil, with differences in OHTs coverage, care-network structure, and integration with PHC.^(18)^

Previous research indicates that dental service use in Brazil remains strongly patterned by socioeconomic and territorial inequalities, with greater utilization in more developed regions and among populations with higher income and educational attainment.^(25)^ Analyses of the Brazilian health system indicate that, despite the universal scope of the SUS, disparities in service provision persist across macro-regions, particularly between the North and Northeast and the South and Southeast.^(26)^ In this context, the uneven recovery observed across Federative Units may reflect the interaction between pandemic-related service disruption and pre-existing vulnerabilities in the organization and delivery of oral health care.

The partial and uneven recovery observed in this study highlights the need for targeted strategies to restore health promotion and prevention activities, which are central to population-level oral disease control.^(27,28)^ Territorial heterogeneity also underscores the importance of policies that address regional inequalities and strengthen service capacity in more vulnerable settings. Consolidating oral health within PHC requires not only restoring service-production levels but also strengthening institutional and service structures to improve resilience to future disruptions.

Several limitations should be considered when interpreting these findings. Because the analysis used aggregated administrative data, its findings depend on the quality of SIA/SUS records and may be affected by variation in data entry, underreporting, coding practices, and reporting consistency across Federative Units and over time. Recorded production reflects the volume of procedures performed but does not directly measure access, service coverage, care quality, unique patients treated, or population oral health needs. The interrupted time-series analysis was based on annual aggregated data with only 11 time points; therefore, its estimates may be unstable and should be interpreted as temporal associations rather than definitive causal effects. Nevertheless, the national dataset and integrated national and Federative Unit-level analyses provide a broad overview of recent trends in recorded PHC dental service production and inform discussion of structural challenges during the post-pandemic period.

## CONCLUSION

Dental service production in Brazilian Primary Health Care declined markedly during the COVID-19 pandemic, against a background of pre-existing downward trends in several indicators. The interrupted time-series analysis identified an immediate reduction in national production rates in 2020, followed by positive post-interruption trends for some indicators. However, recovery from 2022 onward remained partial, heterogeneous, and nonlinear, with seven Federative Units – Ceará, Roraima, Espírito Santo, Tocantins, Pará, Sergipe, and Maranhão – showing mean recovery-period production below the corresponding pandemic-period mean.

Regional variation in recovery patterns may reflect persistent territorial inequalities in the organization and delivery of dental care within the Unified Health System. Together, these findings support continued monitoring of dental service-production indicators, targeted recovery strategies for Federative Units with greater deficits, and sustained policies to finance and strengthen Oral Health Teams within the Family Health Strategy. Such measures may help restore pre-pandemic service-production levels and strengthen the oral health component of a universal, equitable, and resilient Primary Health Care system.
